# Supplementary material for: Construct prognostic models of multiple myeloma with pathway information incorporated
Source: PLoS Comput Biol. 2024 Sep 10;20(9):e1012444. doi: 10.1371/journal.pcbi.1012444 (PMC11414978; doi:10.1371/journal.pcbi.1012444)
Supplement: S1 Table — The values are the mean and standard deviation of 100 duplicated runs. (DOCX) [file pcbi.1012444.s001.docx]

# Table S1, the prediction performance of different methods in internal validation. The values are the mean and standard deviation of 100 duplicated runs.

| paths | C-index | | | | IBS | | | |
| --- | --- | --- | --- | --- | --- | --- | --- | --- |
|  | Grp | GSVA | ssGSEA | Z-score | Grp | GSVA | ssGSEA | Z-score |
| Hall | 0.577(0.042) | 0.550(0.036) | 0.580(0.032) | 0.566(0.031) | 0.192(0.008) | 0.195(0.009) | 0.194(0.009) | 0.196(0.010) |
| Biocarta | 0.565(0.034) | 0.535(0.035) | 0.533(0.038) | 0.552(0.039) | 0.194(0.008) | 0.194(0.009) | 0.197(0.009) | 0.195(0.009) |
| Wiki | 0.573(0.037) | 0.537(0.044) | 0.527(0.034) | 0.549(0.034) | **0.190(0.009)** | 0.197(0.008) | 0.197(0.009) | 0.196(0.008) |
| Pid | 0.552(0.039) | 0.521(0.035) | 0.541(0.036) | 0.529(0.036) | 0.196(0.009) | 0.197(0.009) | 0.196(0.009) | 0.198(0.010) |
| Tft.gtrd | 0.554(0.039) | 0.546(0.037) | 0.573(0.032) | 0.551(0.033) | 0.194(0.008) | 0.195(0.010) | 0.194(0.009) | 0.197(0.009) |
| Tft.legency | 0.552(0.039) | 0.502(0.038) | 0.515(0.036) | 0.530(0.035) | 0.195(0.009) | 0.197(0.009) | 0.197(0.009) | 0.198(0.008) |
| Cgp | 0.581(0.033) | 0.573(0.037) | 0.538(0.037) | 0.561(0.041) | 0.191(0.009) | 0.193(0.009) | 0.198(0.008) | 0.196(0.009) |
| Gobp | 0.550(0.036) | 0.548(0.036) | 0.564(0.037) | 0.571(0.035) | 0.196(0.008) | 0.194(0.009) | 0.193(0.009) | 0.194(0.009) |
| Gocc | 0.588(0.040) | 0.513(0.033) | 0.512(0.028) | 0.551(0.036) | 0.193(0.010) | 0.197(0.009) | 0.196(0.009) | 0.195(0.010) |
| Gomf | 0.563(0.038) | 0.535(0.037) | 0.542(0.038) | 0.575(0.037) | 0.195(0.008) | 0.197(0.010) | 0.196(0.009) | 0.193(0.009) |
| Oncogene | 0.562(0.033) | 0.513(0.030) | 0.543(0.033) | 0.544(0.035) | 0.195(0.008) | 0.196(0.009) | 0.197(0.010) | 0.196(0.009) |
| Cm | 0.557(0.035) | 0.527(0.032) | 0.545(0.039) | 0.541(0.035) | 0.195(0.008) | 0.195(0.010) | 0.196(0.009) | 0.196(0.009) |
| Immune | 0.553(0.036) | 0.536(0.041) | 0.578(0.040) | 0.547(0.035) | 0.195(0.008) | 0.195(0.009) | 0.191(0.009) | 0.197(0.009) |
| Vax | **0.590(0.039)** | 0.536(0.034) | 0.588(0.036) | 0.547(0.036) | **0.190(0.010)** | 0.195(0.009) | 0.192(0.010) | 0.195(0.009) |
| Genes | 0.541(0.037) | | | | 0.195(0.009) | | | |
