# Supplementary material for: Construct prognostic models of multiple myeloma with pathway information incorporated
Source: PLoS Comput Biol. 2024 Sep 10;20(9):e1012444. doi: 10.1371/journal.pcbi.1012444 (PMC11414978; doi:10.1371/journal.pcbi.1012444)
Supplement: S2 Table — (DOCX) [file pcbi.1012444.s002.docx]

# Table S2, the selected pathways of the Vax(grp) model

| **Pathways** | **Population Details** | **Vaccine/Challenge** | **Study Focus** |
| --- | --- | --- | --- |
| QIU_PBMC_HEPTATITIS_B_SURFACE_ANTIGEN_AGE_UNDER50_NON_RESPONDERS_VS_RESPONDERS_28D_U | Hepatitis B surface antigen, Age <50, Non-responders vs. Responders, 28 days | Hepatitis B | Age-related non-responsiveness |
| SOBOLEV_PBMC_PANDEMRIX_AGE_18_64YO_HIGH_VS_LOW_RESPONDERS_MEDIUM_HIGH_ADVERSE_EVENTS_SCORE_1DY_CORRELATED_WITH_TRANSITIONAL_B_CELLS_UP | Pandemrix, Age 18-64, High vs. Low responders, Adverse events score correlated with transitional B-cells, 1 day | Influenza (Pandemrix) | Adverse events & B-cell correlation |
| ZAK_PBMC_MRKAD5_HIV_1_GAG_POL_NEF_AGE_20_50YO_CORRELATED_WITH_CD8_T_CELL_RESPONSE_3DY_NEGATIVE | MRKAD5 HIV-1 gag/pol/nef, Age 20-50, Correlated with CD8 T cell response, 3 days | HIV-1 vaccine | CD8 T-cell response |
| DHIMAN_PBMC_ATTENUVAX_AGE_15_25YO_SUBQ_7_OR_14DY_DN | Attenuvax, Age 15-25, Subcutaneous, 7 or 14 days | Measles (Attenuvax) | Vaccine response |
| PATEL_SKIN_OF_BODY_ZOSTAVAX_AGE_70_93YO_VZV_CHALLENGE_6HR_DN | Zostavax, Age 70-93, VZV challenge, 6 hours | Shingles (Zostavax) | Skin response post-vaccination |
| GARCIA_PINERES_PBMC_HPV_16_L1_VLP_AGE_18_25YO_7MO_CORRELATED_WITH_ANTIBODY_RESPONSE_POSITIVE | HPV-16 L1 VLP, Age 18-25, 7 months, Correlated with antibody response | HPV vaccine | Antibody response |
| ERWIN_COHEN_BLOOD_VACCINE_TC_83_AGE_23_48YO_VACCINATED_VS_CONTROL_2DY_DN | Vaccine TC-83, Age 23-48, Vaccinated vs. Control, 2 days | Venezuelan equine encephalitis | Response to vaccination |
| HOFT_CD4_POSITIVE_ALPHA_BETA_MEMORY_T_CELL_BCG_VACCINE_AGE_18_45YO_ID_7DY_TOP_100_DEG_EX_VIVO_UP | BCG vaccine, Age 18-45, Memory T cell, 7 days, Top 100 DEG ex vivo | Tuberculosis (BCG) | Memory T-cell response |
| ANDERSON_BLOOD_CN54GP140_ADJUVANTED_WITH_GLA_AF_AGE_18_45YO_7DY_UP | CN54gp140 with GLA-AF adjuvant, Age 18-45, 7 days | HIV-1 vaccine candidate | Immune activation |
| HOWARD_NEUTROPHIL_INACT_MONOV_INFLUENZA_A_INDONESIA_05_2005_H5N1_AGE_18_49YO_1DY_DN | Influenza A (H5N1) Indonesia 05/2005, Age 18-49, 1 day | Influenza A (H5N1) | Neutrophil response |
| BUCASAS_PBMC_FLUARIX_FLUVIRIN_CAUCASIAN_MALE_AGE_18_40YO_HIGH_RESPONDERS_1DY_TOP_FUNCTIONAL_NETWORK_POSITIVE | Fluarix/Fluvirin, Caucasian males, Age 18-40, High responders, 1 day | Influenza vaccines | Functional immune network |
| HOWARD_MONOCYTE_INACT_MONOV_INFLUENZA_A_INDONESIA_05_2005_H5N1_AGE_18_49YO_1DY_DN | Influenza A (H5N1) Indonesia 05/2005, Age 18-49, 1 day | Influenza A (H5N1) | Monocyte response |
| ANDERSON_BLOOD_CN54GP140_ADJUVANTED_WITH_GLA_AF_AGE_18_45YO_1DY_DN | CN54gp140 with GLA-AF adjuvant, Age 18-45, 6 hours | HIV-1 vaccine candidate | Immediate immune response |
| HOFT_CD4_POSITIVE_ALPHA_BETA_MEMORY_T_CELL_BCG_VACCINE_AGE_18_45YO_7DY_UP | BCG vaccine, Age 18-45, 7 days (repeat) | Tuberculosis (BCG) | Memory T-cell response |
| FRANCO_BLOOD_SANOFI_PASTEUR_SA_INACTIVATED_INFLUENZA_VACCINE_CORRELATED_WITH_ANTIBODY_RESPONSE_AGE_18_40YO_14DY_NEGATIVE | Sanofi Pasteur SA inactivated influenza vaccine, Age 18-40, 14 days | Influenza vaccine | Antibody response correlation |
| HOFT_CD4_POSITIVE_ALPHA_BETA_MEMORY_T_CELL_BCG_VACCINE_AGE_18_45YO_ID_56D_TOP_100_DEG_AFTER_IN_VITRO_RE_STIMULATION_UP | BCG vaccine, Age 18-45, 56 days, Top 100 DEG after in vitro re-stimulation | Tuberculosis (BCG) | Long-term memory T-cell response |
| KENNEDY_PBMC_DRYVAX_AGE_18_50YO_STIMULATED_VS_UNSTIMULATED_1_TO_48MO_TOP_DEG_UP | Dryvax, Age 18-50, Stimulated vs. Unstimulated, 1 to 48 months | Smallpox (Dryvax) | Long-term immune response |
| OCONNOR_PBMC_MENVEO_ACWYVAX_AGE_30_70YO_7DY_AFTER_SECOND_DOSE_VS_7DY_AFTER_FIRST_DOSE_UP | Menveo (ACWYVax), Age 30-70, 7 days after second vs. first dose | Meningococcal vaccine | Response to booster |
| DHIMAN_PBMC_ATTENUVAX_AGE_15_25YO_SUBQ_7_OR_14DY_UP | Attenuvax, Age 15-25, Subcutaneous, 7 or 14 days (repeat) | Measles (Attenuvax) | Immediate immune response |
