# Supplementary material for: Construct prognostic models of multiple myeloma with pathway information incorporated
Source: PLoS Comput Biol. 2024 Sep 10;20(9):e1012444. doi: 10.1371/journal.pcbi.1012444 (PMC11414978; doi:10.1371/journal.pcbi.1012444)
Supplement: S1 Fig — P-values were obtained using a corrected resampled t-test and were not adjusted for multiple testing. The lower triangular part of the matrix represents the significance for IBS, while the upper triangular part represents the significance for the C-index. (DOCX) [file pcbi.1012444.s004.docx]

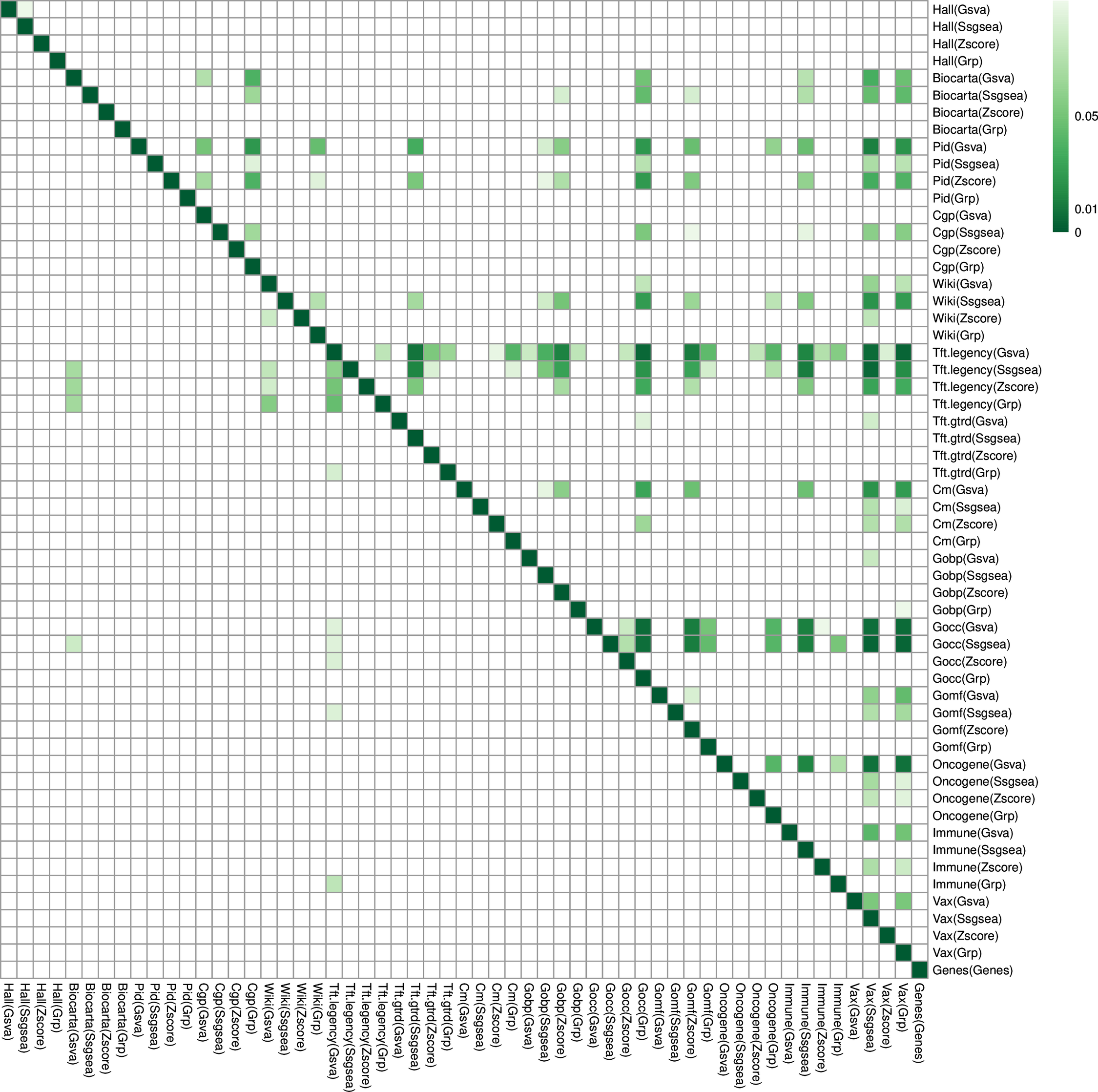


Figure S1, Statistical significance for pairwise comparisons. P-values were obtained using a corrected resampled t-test and were not adjusted for multiple testing. The lower triangular part of the matrix represents the significance for IBS, while the upper triangular part represents the significance for the C-index.
