# Supplementary material for: Construct prognostic models of multiple myeloma with pathway information incorporated
Source: PLoS Comput Biol. 2024 Sep 10;20(9):e1012444. doi: 10.1371/journal.pcbi.1012444 (PMC11414978; doi:10.1371/journal.pcbi.1012444)
Supplement: S2 Fig — The survival curve of the high and low risk groups predicted by different models. The median of linear predictors was used as the cutoff. *, the model was trained with that data. (DOCX) [file pcbi.1012444.s005.docx]

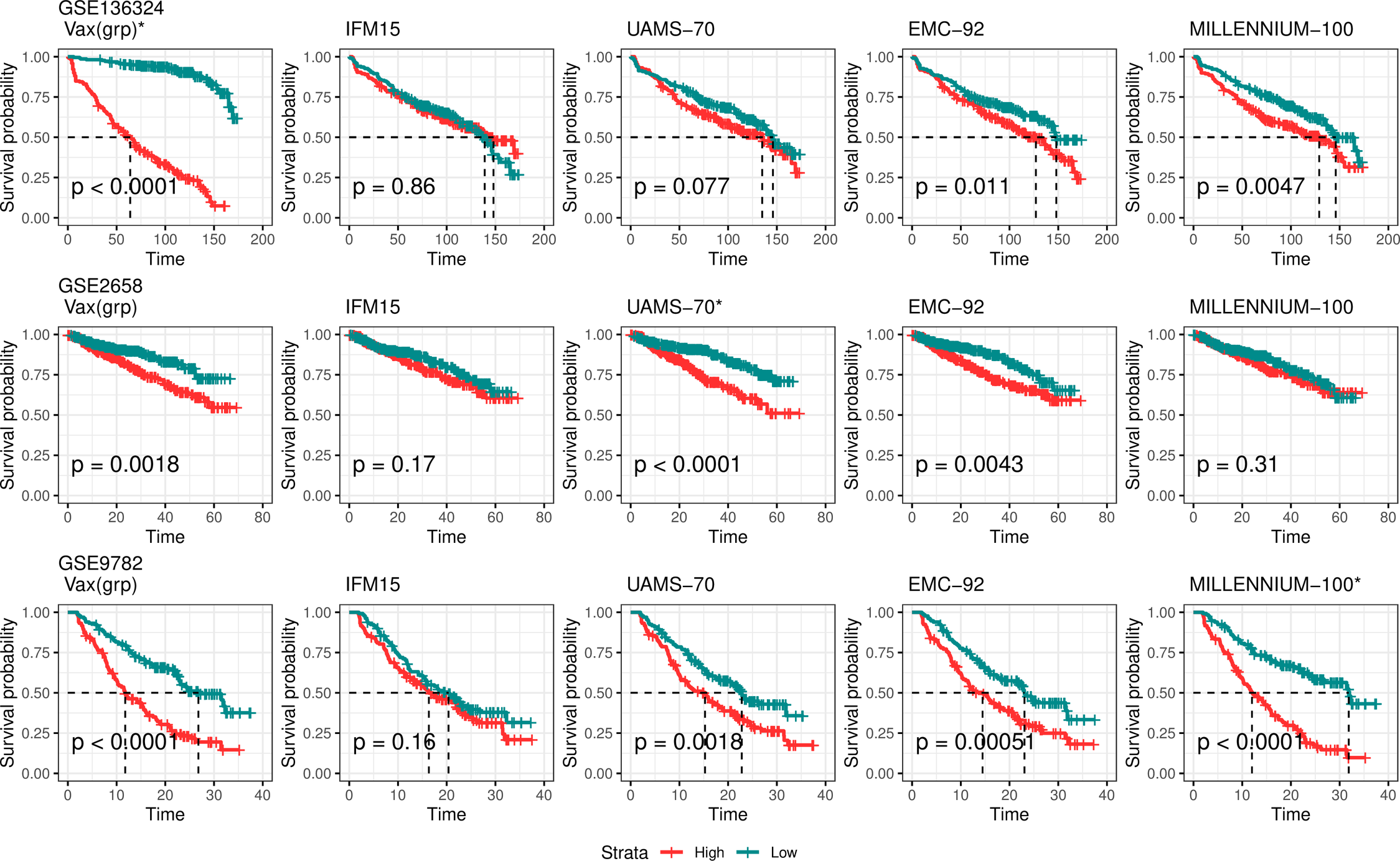


Figure S2, Kaplan-Meier Plot. The survival curve of the high and low risk groups predicted by different models. The median of linear predictors was used as the cutoff. *, the model was trained with that data.
