# Supplementary material for: Construct prognostic models of multiple myeloma with pathway information incorporated
Source: PLoS Comput Biol. 2024 Sep 10;20(9):e1012444. doi: 10.1371/journal.pcbi.1012444 (PMC11414978; doi:10.1371/journal.pcbi.1012444)
Supplement: Attachment — Submitted filename: Response to reviewers.docx [file pcbi.1012444.s006.docx]

**Point-by-Point Response to Reviewers’ comments for "Construct Prognostic Models of Multiple Myeloma with Pathway Information Incorporated" by Wang et al.**

**Comment from Editor**

Thank you very much for submitting your manuscript "Construct Prognostic Models of Multiple Myeloma with Pathway Information Incorporated" for consideration at PLOS Computational Biology.

As with all papers reviewed by the journal, your manuscript was reviewed by members of the editorial board and by several independent reviewers. In light of the reviews (below this email), we would like to invite the resubmission of a significantly-revised version that takes into account the reviewers' comments.

The pathway-based approach was found interesting by all the reviewers, while pointing out shortcomings that, if addressed, may significantly strengthen the paper. Please address these suggestions as best as you can.

Reviewer 2 noted that a discussion related to the computational complexity of the presented model. and how it compares to existing models, considering its accuracy, is missing. This is an important point to address, as it has a direct impact on clinical translation, as noted also by Reviewer 3.

We cannot make any decision about publication until we have seen the revised manuscript and your response to the reviewers' comments. Your revised manuscript is also likely to be sent to reviewers for further evaluation.

Response: We appreciate these constructive comments. Following these suggestions, we added a new subsection in the Results section to demonstrate the sources of computational complexity in the presented model (Page 26, lines 3-16). For comparison with existing models, we performed statistical tests between the new models and competitor models, demonstrating the significance of better predictive performance of our models (Table 4). Regarding clinical translation, we expanded our discussion on the benefits of the pathway modeling strategy in advancing biological research and prognostic predictions (Page 28, lines 12-23; Pages 29, 1-9), as well as the challenges of clinical translation (Pages 29, Lines 11-22). We further discussed the limitations and future direction of this study (Page 30, lines 6-21). (All the page and line numbers in the response letter are based on the version with changes tracked. They may be different in the clean version)

**Reviewer #1**:

The manuscript provides insights into the use of pathway information for developing prognostic models in Multiple Myeloma. However, I have three minor points for revision:

1) Clarity on Batch Correction Application: Please specify where batch correction was applied in your analyses. Your manuscript mentions adjusting batch effects for the training data of competitor models, when applicable (Page 15, Line 15). Clarifying exactly where batch correction was applied (and where it was not) would greatly aid in understanding the methodological rigor and ensuring the robustness of your comparative analysis.

Response: Thanks for the constructive suggestion. Out of the four competitor models, batch effects correction was required for the MILLENNIUM100, EMC-92, and IFM15 models as they need the absolute expression matrices as input. The new datasets (GSE2658 and GSE9782) were adjusted to be comparable to the training data of these models. The parametric empirical Bayes frameworks, namely comBat, implemented in the sva package(version 3.46.0) was used for batch effect correction (Johnson et al. 2007). The UAMS-70 model accepts the relative expression of upregulated and downregulated genes as inputs; hence, batch effects correction is not necessary. **We improve our draft by including the information regarding the batch correction (Page 16, lines 15-20). (All the page and line numbers in the response letter are based on the version with changes tracked. They may be different in the clean version)**

2) Pathway Scoring Justification: Could you provide a more detailed motivation for your choice of pathway scoring methods? While the comparison with gene-based models is clear, understanding the specific rationale behind selecting these pathway scoring methods would enhance the context and depth of your study.

Response: We thank the reviewer for this valuable feedback. We have provided more justifications for the choice of pathway scoring methods. The present study included three pathway scoring methods: ssGSEA, GSVA, and Z-score. Our rationale behind the choice of those three methods is as below:

First, we prefer to include the pathway-scoring methods that are widely recognized in the field of bioinformatics. For example, ssGSEA and GSVA have been cited nearly 50,000 and 10,000 times, respectively, indicating their widespread use and acceptance. Z-score was selected for its simplicity.

Additionally, each of these methods has distinct features. For instance, ssGSEA relies on rank information, and its calculation does not depend on background samples. In contrast, GSVA and Z-score are representative methods that depend on the distribution of all samples in the cohort, and therefore require a reference cohort if scores need to be calculated for new samples. Other scoring methods, such as PCA and Pathifier, were not used in the current study for various reasons. PCA is very sensitive to extreme values and choosing which PC for pathway score is not a trivial task. Pathifier is computationally intensive and requires parameter tuning and therefore not suitable for large-scale benchmarking and clinical application. In addition, Pathifier has also been shown to be sensitive to outliers.

**We have revised our manuscript to include this information in the Methods section (Page 9, lines 21-23; Page 10, lines 1- 7).**

3) The sentence in Page 5, Line 9 seems to have grammatical issues

Response: Thanks for pointing out the grammatical issues. We have revised this sentence as below:

However, few of them have been applied in practice for several reasons, including insufficient predictive accuracy, lack of generalization to the whole population, and poor model reporting.

**Reviewer #2:**

In this paper, the authors explore novel prognostic modeling approaches for Multiple Myeloma (MM), focusing on the utilization of pathway information rather than conventional gene-level data. Two primary strategies are assessed: the pathway score method and group lasso with pathway information. The study tests various methods for calculating pathway scores and integrates this information into predictive models, using microarray data (GSE136324) for MM. The best-performing model, based on group lasso incorporating Vax pathway information (Vax(grp)), demonstrates superior predictive accuracy compared to gene-based models and previously published approaches, both in internal and external validations. Despite the manuscript presenting high levels of accuracy, a good level of English and providing a valuable contribution to current research, it needs some improvements:

1, Improve the introductory section of the manuscript by highlighting and listing both the “contributions” points of this research and the work's limitations;

Response: We thank the reviewer for the suggestions. **We added a brief description of the significance of our study in the introduction (Page 6, lines 4-7). A more detailed discussion of the contributions and limitations of our work has been added to the discussion (Page 30, lines 1-20) in the revised manuscript. (All the page and line numbers in the response letter are based on the version with changes tracked. They may be different in the clean version.)**

At the end of the Introduction section, we have added the sentences as below:

Our benchmark results demonstrated clear advantage of pathway-based models in clinical outcome prediction. Our computational workflow can also serve as a comprehensive guidebook for future studies where prior knowledge needs to be incorporated into predictive models.

At the end of the Discussion section, we have added:

The primary contribution of this study is our proposal to incorporate existing knowledge into model building. Data-driven model building, such as gene models, is challenging due to the need for a large sample size. However, it is difficult to obtain enough samples, especially in high-dimensional setups. Building pathway models is guided by existing knowledge, which can reduce the sample size requirement and allow us to build on previous research findings, rather than starting from scratch.

We are also aware of the limitations of the current study. Firstly, we only covered limited numbers of pathway scoring methods and prediction models. For example, we only used Lasso regression for predicting clinical outcomes due to its simplicity and interpretability. However, other machine learning models, such as Random Forest, XGBoost or deep learning models may further improve the prediction accuracy. Better pathway scoring methods, such as those considering the direction of differential expression, may also benefit our model. Secondly, while our model proposed Vax, a computational pathway database of vaccination-related gene expression changes, as the best source of pathway knowledge, the evidence of biological relevance is currently unclear, which awaits experimental validation. Last but not least, we only tested our approaches in Multiple Myeloma. To make our method more generalizable, a border benchmark study in the future is required to test whether the pathway-based models improve patient stratification in our cancer types and which knowledge database should be used for a specific disease. Including disease-specific clinical parameters, such as tumor stages, genomic background and so on, may provide additional power to our prediction model. Nevertheless, our current study provides a valuable guidebook for such benchmark studies in the future.

2, While the study presents promising results, the generalizability of the findings to other types of hematological diseases remains to be explored. Please discuss them in the possible limitations of the work or as possible future tasks;

Response: We thank the reviewer for the helpful comments. We have included the discussion of the generalizability of our results as well as the possible future work in the last paragraph in the Discussion section, as detailed in the response to Question 1.

3, Considerating the complexity of Pathway Analysis, please discuss how the complexity of these models may affect their translation from research to clinical practice, considering the computational load that may be placed on clinical systems by offering potential solutions or areas for future development that may help reduce the computational burden, such as algorithmic improvements or the use of more efficient computing frameworks. So, explain better the complexity of the proposed framework, which might make it difficult to implement in real-world scenarios.

Response: We thank the reviewer for the comment. The computational complexity of implementing these models in clinical practice lies in the data preparation procedure. For models based on pathway scores, data preparation involves batch effects adjustment, missing data imputation (if necessary), and pathway score computation. For models based on group lasso, data preparation includes batch effects adjustment and missing data imputation (if necessary). **We have added a subsection in the Results section to illustrate the computational complexity and detail the time consumption for the proposed models in Table 6 (Page 26, lines 3-16).** In general, without missing values, the prediction (including batch effects correction) can be achieved within seconds. Overall, compared to the time required to generate gene expression data for new patients, which may take days to weeks, the time required for running our bioinformatic pipeline is negligible and therefore does not introduce significant hurdles for clinical translation.

**Table 6, model computation cost when applied to new data (unit, seconds)**

|  | GSE9782 | | | GSE2658 | | |
| --- | --- | --- | --- | --- | --- | --- |
|  | Vax(grp) | Vax(ssgsea) | Genes | Vax(grp) | Vax(ssgsea) | Genes |
| Batch Effects Correction | 0.884 | 0.885 | 0.867 | 1.196 | 1.656 | 1.667 |
| Path Score Calculation | / | 2.629 | / | / | 4.363 | / |
| Prediction | 0.002 | <0.001 | <0.001 | 0.004 | <0.001 | <0.001 |
| Miss Imputation | 254.421 | 265.482 | 253.443 | 506.587 | 497.131 | 499.474 |

Notes: Models were executed using R Software (version 4.3.0) on the Windows 11 (x64) operating system. The computer was equipped with an AMD Ryzen 9 7950X 16-core Processor and four 16GB RAM modules. No parallel running was used. Additionally, 1% of missing values were randomly generated.

4, Please to include specific statistical tests to quantify the effectiveness of the proposed method and simulation;

Response: We thank the review for raising this important issue. For the internal validation, we presented the 95% confidence interval of mean accuracy (Mean ± 1.96*SE) as error bars instead of the median and intervals in the revised figure (Figure 2) to better indicate the uncertainty. In addition, we performed a pairwise comparison based on a corrected resampled t-test. Vax(grp) demonstrated the highest accuracy in prediction as indicated by C-index and IBS, although no significant difference was observed when compared with some of the competitive models (Figure S1). Nevertheless, Vax(grp) was still the most promising model based on mean accuracy and therefore was chosen for further comparison with external data **(Page 20, lines 19-21; Page 21, lines 1-3).**

For the external validation, we added the 95% confidence interval based on 1000 Bootstrap replicates (Table 3 and Table 4). We also examined the statistical significance between these models in Table 3 and compared the proposed methods (Vax, grp) with existing methods in Table 4. The proposed model demonstrated significantly superior prediction accuracy compared to existing models in the external validation **(Page 21, lines 11-12; Page 22, 10).**

**Figure 2, the prediction performance of pathways-based / gene-based models in internal validation.** Upper, prediction measured with C-index. Lower, IBS. The dots and error bars represent mean ± 1.96*SE (standard error).

**Table 3, the prediction performance of selected models in two external data.** Bold, the best one for each measure. Values in the parentheses represent the 95% confidence interval, derived from 1000 bootstrap samples. Vax(grp), Vax(ssgsea), and Genes models were significantly different from each other (adjusted P < 0.001). P values were adjusted using the Bonferroni method.

|  | GSE9782 | | GSE2658 | |
| --- | --- | --- | --- | --- |
|  | C-index | IBS | C-index | IBS |
| Vax(grp) | **0.653(0.607,0.700)** | **0.335(0.300,0.369)** | **0.653(0.588,0.722)** | **0.144(0.121,0.160)** |
| Vax(ssgsea) | 0.632(0.581,0.681) | 0.347(0.322,0.394) | 0.604(0.536,0.663) | 0.151(0.133,0.174) |
| Genes | 0.618(0.569,0.668) | 0.349(0.314,0.385) | 0.625(0.554,0.694) | **0.144(0.124,0.163**) |

**Table 4, compare the proposed model with previous models using the C-index.** *, models were fitted with corresponding data (training data). Bold, the best one for each data. Values in the parentheses represent the 95% confidence interval, derived from 1000 bootstrap replicates. Vax(grp) was significantly different from the competitor models in all three data (all P < 0.001).

|  | GSE136324 | GSE2658 | GSE9782 |
| --- | --- | --- | --- |
| EMC-92 | 0.567(0.520, 0.610) | 0.634(0.562, 0.706) | 0.629(0.578, 0.680) |
| UAMS-70 | 0.560(0.514, 0.602) | 0.658(0.588, 0.726)* | 0.606(0.553, 0.656) |
| IFM15 | 0.508(0.465, 0.553) | 0.568(0.507, 0.628) | 0.563(0.510, 0.615) |
| MILLENNIUM-100 | 0.582(0.538, 0.627) | 0.575(0.506, 0.640) | 0.682(0.633, 0.726)* |
| Vax(grp) | 0.854(0.829, 0.878)* | 0.653(0.588, 0.722) | 0.653(0.607, 0.700) |

5, This study could benefit from comparisons with the latest deep learning or machine learning techniques in prognostic modeling. Please to discuss them.

Response: We appreciate the suggestion from the reviewer. We agree that other machine learning techniques, such as random forest, gradient boosting, SVM, and so on, or deep learning methods, can also be used for incorporating pathway information into prediction models and may have better performance than simple multiple linear regression, However, we prefer multiple linear regression with Lasso penalty because of its simplicity and interpretability. More sophisticated machine learning or deep learning methods typically require large sample sizes and often face challenges in model interpretability, and therefore not ideal for clinical applications Nevertheless, our current study in a simple context demonstrates the principles and benefits of incorporating pathway information in outcome prediction and opens up the possibility for building and testing more advanced machine learing/AI methods. **We discussed this on Page 9, Lines 1-10 (Method section); Page 30, lines 6-11 (Discussion section).**

6, Given the complexity of pathway-based models, the study could delve deeper into the interpretability of these models, which is crucial for clinical acceptance and application. How do the authors overcome and discuss that difficulty?

Response: We thank the reviewer for this comment. We extended our discussion of tour model interpretability in the revised manuscript. The gene-based models select individual genes as predictors. Due to the collinearity of the gene expression profile (many genes show high correlation with each other) and the regularization (e.g. Lasso penalty) during feature selection, the genes selected in the model are not readily interpretable as genes correlated with the selected ones may be randomly dropped by the model, but they may have important biological functions. On the other hand, the pathway-based models first summarize the individual gene expression changes on pathway activity level, which can be considered as a pathway-guided dimension reduction and denoise process. Therefore, the pathway-based models are more stable and interpretable than the gene-based models. For example, in our study, the two most predictive models are those based on immune-related pathways, which is consistent with the fact that Multiple Myeloma is a cancer of plasma cells, which produce antibodies and therefore are a critical component of the adaptive immune system.

The Vax(grp) model selected 20 different pathways from the Vax pathway database (Table S2 in Supporting Information). These pathways were derived from the Human Immunology Project Consortium describing human transcriptomic immune responses to vaccinations. Many of these selected pathways have been validated by previous studies. For example, functions involved in the hepatitis B virus vaccine response were a predictor in our model. Many studies documented that virus infection increases the risk of Multiple Myeloma (Huang et al. 2012; Li et al. 2016; Su et al. 2019; Tsukune et al. 2019). In addition, more selected vaccine response including HIV, Measles, Shingles, Human papillomaviruses (HPV), Tuberculosis, and Influenza (Yee et al. 2001; Dingli et al. 2009; Packiriswamy et al. 2020; Ahmadinejad and Mansouri 2013; Ahn et al. 2013; Tsai et al. 2017; Rocha et al. 2023; Hahn et al. 2015; Thompson et al. 2023).

**We detailed the pathways in Table S2 and discussed them in the discussion section (Page 28, Lines 12-23; Page 29, Lines 1-9).**

7, The transition from a research setting to clinical implementation requires further validation, especially in terms of how these models might influence therapeutic decision-making in real-world scenarios. Please discuss that;

Response: We thank the reviewer for this suggestion. We added a brief discussion, as below, regarding the potential clinical translation of our model in the **Discussion Section (Page 29, lines 11-22).**

Although numerous models have been proposed, few have transitioned into clinical applications. This gap largely arises from insufficiently transparent reports and a lack of neutral comparison. For this sake, we improve the reproducible documentation of our models, including information on the patient populations, training data, validation data, and model coefficients. However, we acknowledge that significant work remains to be done before these models can be implemented in clinical settings or impact therapeutic decision-making. First of all, more independent test datasets are needed to validate the robustness and generalizability of our current model. Secondly, our model can also benefit from further optimization, such as including clinical parameters as additional predictors as well as implementing other machine learning/AI methods for deriving pathway scores and prediction. Most importantly, any models still need to undergo rigorous clinical trials before the approved by regulatory bodies, such as the FDA in the United States or the EMA in Europe, and the eventual applications in routine clinical practice.

8, The "Conclusions" section should be expanded to offer a more comprehensive view and analytical perspective on future prospects by considering and adding a comparative analysis.

Response: We thank the reviewer for the constructive comment. We agree that the Conclusion section in the previous version of our manuscript was too shallow and lacked prospects. Considering all the very constructive feedback from the reviewer above, including 1) the clarification of the contribution/limitation of our model; 2) a detailed discussion of complexity and interpretability, 3) a comparison with other machine learning/AI methods, 4) clinical translation and 5) future prospects by considering and adding a comparative analysis, we decided to overhaul our "Discussion" to address all those points and remove the previous "Conclusion" section. We believe the structure of our manuscript will be more clear and the discussion section will be more cohesive in this way.

**Reviewer #3**:

The paper from Wang et al is of interest and describe a new method to infer and interpret the pathway analysis and how they can explain cancer development and biology.

The paper is well written and despite the high complexity of the applied model it is easy to understand.I have only a concern based on the idea that this model could be applied for decision making and also could outperformed compared with the previous models.

This could be right from a technical point f view, which is the scope of the work, but for really say that outperformed it would be nice if the authors could provide some survival predictions based on they new model.

Response: We thank the reviewer for the positive feedback on our work and the constructive comments. As suggested, we further performed survival prediction analysis and illustrated the performance of our new model to competitor models in the new data using Kaplan-Meier plots (see Figure S2 in the Supporting Information). The proposed model outperformed the competitor models illustrated by a higher capacity in discriminating the high- and low-risk groups across the three data. Of note, GSE136324, GSE2658, and GSE9782 were the training data for Vax(grp), UAMS-70, and MILLENNIUM-100, respectively, which may account for their enhanced predictive accuracy in these datasets. A more comprehensive comparison was added in Table 4, where the proposed model (Vax(grp)) exhibited more accurate prediction than the rest based on C-index. **We added a Figure in the Supporting Information (Figure S2) and revised our manuscript on Page 22, lines 10-13. In revised Figure 2, Table 3, and Table 4, we also added uncertainty metrics to indicate the significance of our model superiority. (All the page and line numbers in the response letter are based on the version with changes tracked. They may be different in the clean version.)**

Figure S2, Kaplan-Meier Plot. The Median of linear predictors was used as the cutoff. *, the model was trained with that data.

The knowledge of how a cancer work (deregulated pathways) is absolutely more important rather than the knowledge of a some gene deregulation. Nevertheless, the application of other scores, even if gene-based, has clinical and prognostic impact. Is also the case for the author's model?

Response: We thank the reviewer for the insightful comments. We agree that the application of other scores and gene-based models do have clear clinical application potential, due to their simplicity. In our study, we would like to emphasize that, according to our results, we argue the pathway-based models have higher interpretability and robustness that can facilitate clinical translation due to below reasons:

As pointed out by the reviewer, a key advantage of the pathway-based modeling strategy is its ability to predict prognostics using the activities of biological functions. This approach underscores the idea that cancer development involves the disorder of relevant biological functions rather than the alteration of a few specific genes. The resulting model not only serves as a prognostic tool but also highlights the functions relevant to the disease, thanks to the sparsity property of the Lasso regression. On the other hand, the gene-based models select individual genes as predictors. Due to the collinearity of the gene expression profile (many genes show high correlation with each other) and the regularization (e.g. Lasso penalty) during feature selection, the genes selected in the model are not readily interpretable as genes correlated with the selected ones may be randomly dropped by the model but they may have important biological functions. Furthermore, the pathway-based models first summarize the individual gene expression changes on pathway activity level, which can be considered as a pathway-guided dimension reduction and denoise process. Therefore, the pathway-based models are more stable and interpretable than the gene-based models. For example, in our study, the two most predictive models are those based on immune-related pathways, which is consistent with the fact that Multiple Myeloma is a cancer of plasma cells, which produce antibodies and therefore are a critical component of the adaptive immune system.

We agree that the generalizability of our approach needs validation in other hematological malignancies and cancer types. This is particularly crucial for less well-studied diseases, where prior knowledge may not provide sufficiently accurate information for pathway modeling. In such cases, alternative scores or gene-based models might be more appropriate.

**We have added the above discussion the revised manuscript (Page 27, line 14-20; Page 28, line 12-23; Page 29, line 1)**

Publication bibliography

Ahmadinejad, Zahra; Mansouri, Seddighe (2013): Tuberculosis of spine as the first clinical presentation of Multiple Myeloma: a case report. In *Tehran University Medical Journal* 71 (4).

Ahn, Jae-Sook; Rew, Sung Yoon; Yang, Deok-Hwan; Jung, Sung-Hoon; Kang, Seung-Ji; Kim, Mi-Young et al. (2013): Poor prognostic significance of Mycobacterium tuberculosis infection during bortezomib-containing chemotherapy in patients with multiple myeloma. In *Blood research* 48 (1), p. 35.

Amin, Samirkumar B.; Yip, Wai-Ki; Minvielle, Stephane; Broyl, Annemiek; Li, Yuedan; Hanlon, Bret et al. (2014): Gene expression profile alone is inadequate in predicting complete response in multiple myeloma. In *Leukemia* 28 (11), pp. 2229–2234.

Dingli, David; Offord, Chetan; Myers, Rae; Peng, Kah-Whye; Carr, Thomas W.; Josic, Kresimir et al. (2009): Dynamics of multiple myeloma tumor therapy with a recombinant measles virus. In *Cancer gene therapy* 16 (12), pp. 873–882.

Hahn, Michael; Schnitzler, Paul; Schweiger, Brunhilde; Kunz, Christina; Ho, Anthony D.; Goldschmidt, Hartmut; Schmitt, Michael (2015): Efficacy of single versus boost vaccination against influenza virus in patients with multiple myeloma. In *Haematologica* 100 (7), e285.

Huang, Beihui; Li, Juan; Zhou, Zhenhai; Zheng, Dong; Liu, Junru; Chen, Mei (2012): High prevalence of hepatitis B virus infection in multiple myeloma. In Leukemia & Lymphoma 53 (2), pp. 270–274.

Johnson, W. Evan; Cheng Li; and Ariel Rabinovic. (2007): Adjusting batch effects in microarray expression data using empirical Bayes methods. Biostatistics 8.1, pp. 118-127.

Kaneko, M.; Kanda, Y.; Oshima, K.; Nannya, Y.; Suguro, M.; Yamamoto, R. et al. (2002): Simple prognostic model for patients with multiple myeloma: a single-center study in Japan. In *Annals of hematology* 81 (1), pp. 33–36.

Li, Y.; Bai, O.; Liu, C.; Du, Z.; Wang, X.; Wang, G.; Li, W. (2016): Association between hepatitis B virus infection and risk of multiple myeloma: a systematic review and meta‐analysis. In *Internal Medicine Journal* 46 (3), pp. 307–314.

Liang, Ruqing; Wang, Meng; Zheng, Guizhi; Zhu, Hua; Zhi, Yaqin; Sun, Zongwen (2018): A comprehensive analysis of prognosis prediction models based on pathway‑level, gene‑level and clinical information for glioblastoma. In *International journal of molecular medicine* 42 (4), pp. 1837–1846.

Meinshausen, Nicolai; Bühlmann, Peter (2010): Stability selection. In *Journal of the Royal Statistical Society: Series B (Statistical Methodology)* 72 (4), pp. 417–473.

Packiriswamy, Nandakumar; Upreti, Deepak; Zhou, Yumei; Khan, Rehan; Miller, Amber; Diaz, Rosa M. et al. (2020): Oncolytic measles virus therapy enhances tumor antigen-specific T-cell responses in patients with multiple myeloma. In *Leukemia* 34 (12), pp. 3310–3322.

Perrot, Aurore; Lauwers-Cances, Valérie; Tournay, Elodie; Hulin, Cyrille; Chretien, Marie-Lorraine; Royer, Bruno et al. (2019): Development and validation of a cytogenetic prognostic index predicting survival in multiple myeloma. In *Journal of Clinical Oncology* 37 (19), p. 1657.

Rocha, Luís A.; Silva, Luciana; Miranda, João; Soares, Maria Inês; Moreira, Albina (2023): Meningococcal Septic Oligoarthritis: An Unusual Presentation Revealing Concurrent Multiple Myeloma. In *Cureus* 15 (12).

Su, Tung‐Hung; Liu, Chun‐Jen; Tseng, Tai‐Chung; Chou, Shih‐Wan; Liu, Chen‐Hua; Yang, Hung‐Chih et al. (2019): Chronic hepatitis B is associated with an increased risk of B‐cell non‐Hodgkin's lymphoma and multiple myeloma. In *Alimentary Pharmacology & Therapeutics* 49 (5), pp. 589–598.

Sun, Chen; Li, Hongyang; Mills, Ryan E.; Guan, Yuanfang (2019): Prognostic model for multiple myeloma progression integrating gene expression and clinical features. In *Gigascience* 8 (12), giz153.

Thompson, Michael A.; Boccadoro, Mario; Leleu, Xavier; Vela-Ojeda, Jorge; van Rhee, Frits; Weisel, Katja C. et al. (2023): Rates of influenza and pneumococcal vaccination and correlation with survival in multiple myeloma patients. In *Clinical Lymphoma Myeloma and Leukemia* 23 (3), e171-e181.

Tsai, Chun-Kuang; Huon, Leh-Kiong; Ou, Shuo-Ming; Kuan, Ai-Seon; Yeh, Chiu-Mei; Lee, Yu-Ting et al. (2017): Risk and impact of tuberculosis in patients with multiple myeloma. In *Leukemia & Lymphoma* 58 (11), pp. 2598–2606.

Tsukune, Yutaka; Sasaki, Makoto; Komatsu, Norio (2019): Reactivation of hepatitis B virus in patients with multiple myeloma. In *Cancers* 11 (11), p. 1819.

Yee, T. T.; Murphy, K.; Johnson, M.; Abdalla, S. H.; Patton, G. S.; Lee, C. A.; Mehta, AB (2001): Multiple myeloma and human immunodeficiency virus‐1 (HIV‐1) infection. In *American journal of hematology* 66 (2), pp. 123–125.

Yu, Bin (2013): Stability.
